# Establishment of Solid–Liquid–Solid Double-Layer Model of Silicon–Aluminum Phase in Mixed-Medium and Synergistic Stabilization Experimental Study

**DOI:** 10.3390/ma18071523

**Published:** 2025-03-28

**Authors:** Jiaming Zou, Weijun Yang, Jianyu Yang, Peng Shen

**Affiliations:** School of Civil Engineering, Changsha University of Science and Technology, Changsha 410014, China; yyyaozhijian@163.com (W.Y.); 18229816192@163.com (P.S.)

**Keywords:** silicon–aluminum, electrical double layer, consolidation, solid waste, experiment

## Abstract

The issue of low resource utilization rate and high treatment cost in the disposal of construction waste and solid waste was a challenging problem. In order to seek a synergistic and efficient treatment method, based on the similarity of microstructural characteristics between clay, solid waste, and lithium slag particles, a dual-layer theory and model was used to conduct adaptive analysis at the electrochemical level, studying the solid–liquid–solid dual-layer theoretical model suitable for silicon–aluminum-phase materials. At the same time, the phenomenon of particle interface contact and the influence mechanism of ion adsorption on the surface of particles in the liquid phase were discussed, analyzing the ion selection mechanism for regulating the dual-layer of silicon–aluminum-phase materials and studying the method of clay-modified stabilization based on solid waste. Further laboratory tests and microscopic analyses were conducted to determine the engineering properties of the soil stabilized by the clay–solid waste synergistic stabilization and verified the effectiveness of the method. The research results showed that the trial soil stabilized by the theoretical model guidance was significantly stronger in unconfined compressive strength (1.44 MPa at 28 days) than the undisturbed clay (0.26 MPa at 28 days), and the scanning electron microscope (SEM) microscopic analysis results showed that the microscopic morphology of the modified stabilized soil specimen was tightly woven with a high-strength network-like structure. The research provided a theoretical basis and experimental reference for the synergistic treatment and resource utilization of waste soft soil and solid waste engineering problems.

## 1. Introduction

Today’s construction projects could not be separated from land excavation, due to the problem of soil quality in the construction area, the excavation of land inevitably produced a large amount of weak, unstable, and low-activity waste soil [1,2]. The transportation and replacement of soil generated huge cost problems, and the waste land resources had not been fully utilized. At the same time, with the continuous production of industrial manufacturing, the reality of the accumulation of large quantities of solid waste, the problem of low resource utilization rate, and high processing cost were increasingly prominent [3]. People were trying to seek a variety of technical solutions and measures [4], of which soil stabilization technology had unique additive and modification technology features, especially in the trial of alkali activation and additive modification at the level [5]. However, faced with the thorny problem of low resource utilization rate and high treatment cost for waste soil and solid waste, the existing research still did not find a suitable method [6,7]. For the huge resource utilization status encountered in construction projects, the existing technology was limited by processing means, mechanical equipment, and process conditions and could not achieve a simple and efficient mass treatment.

**Figure 1:**
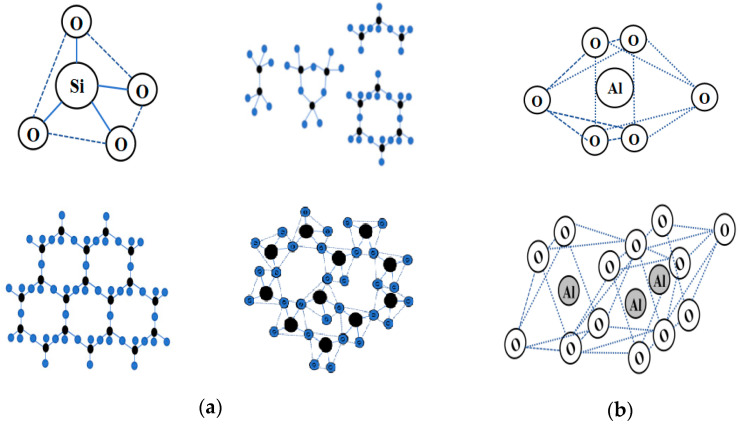
A schematic diagram of the molecular structure of the Si-Al-phase precursor: (**a**) scheme of silicon oxygen tetrahedron and partially stacked forms(black refers to silicon elements, blue refers to oxygen elements); (**b**) aluminum oxygen octahedral and partially stacked forms.

Currently, the excavated waste soil was mostly composed of clay, and at the microscopic level, the microstructural features of clay molecules were similar to those of most rock minerals in nature, which were mostly composed of silicon tetrahedra and aluminum tetrahedra or octahedra [8,9]. It was worth noting that the mineral composition of many waste mine tailings, such as solid waste, was similar to that of silicon–aluminum phases, and some industrial solid waste, such as slag and lithium slag, for example, lithium slag, were composed of silicon–aluminum-phase molecular structures [10,11]. Because of the variable crystal structure characteristics of the silicon–aluminum-phase units, the difference between soil particle minerals and solid waste ore materials lay in the different stacking patterns of the tetrahedra and octahedra, as shown in Figure 1.

Due to the similar silicon–aluminum-phase molecular structure of clay particles and some solid waste particles (such as lithium slag) [12,13], a material basis for their synergistic treatment was provided. In recent years, some researchers have made active exploration in finding effective, safe, and environmentally friendly industrial solid waste materials for soil stability research. Some alkaline industrial solid waste has been proven to be effective in improving soil performance [14,15]. In terms of composition, the primary chemical components of these industrial solid wastes (such as slag and lithium slag) were SiO_2_, Al_2_O_3_, CaO, and MgO, demonstrating high pozzolanic activity and showing great potential in soil solidification [14]. To confirm the compositional characteristics, a more detailed analysis of main chemical components of soft soil and lithium slag was performed using XRD on the experimental material selection in this research. Meanwhile, several studies have confirmed the efficient and feasible migration of these materials into the soil. Compared with traditional cement materials, some silica and aluminum waste materials show better permeability resistance and environmental protection when used as paving blocks or combined with soil [16,17]. Furthermore, the research barriers and theoretical applicability of the synergistic treatment method still existed, mainly concentrated in two aspects of a and b, as follows:

a. A large number of studies continuously tried to study various solidification agents and multiple types of additives and initiators. The research feedback produced a large number of trials that were still trying out formulas and methods. Although some alkali cations were beneficial in enhancing the potential activity of the silicon–aluminum-phase materials and improving material performance [18], the cost of the formula was difficult to control, and the grasp of dosage and adaptability was in an empirical state, lacking a guiding theory.

b. According to the reports of existing studies, thermal treatment, chemical modification, physical mechanical processing, and combined treatment methods were tried to seek effective treatment solutions from the basic understanding of physical, chemical, and hydration perspectives [3,4,19]. However, these methods had many types of unconventional and large-scale processing limitations, such as considering environmental, condition, and economic factors in general engineering applications, and could not achieve mechanical grinding or high-temperature furnace heating and high cost additions. The traditional theoretical thinking method had limited adaptability.

To address the problems mentioned above, it was essential to continuously strive to reveal the interface contact situation between waste solid particles and soil in a solvent environment and the influence mechanism of ion adsorption on the surface of soil particles in the liquid phase. Therefore, it was necessary to introduce the concept of double layer on the interface of silicon–aluminum phases in the mixed medium because the electrochemical phenomena of the double layer was closer to the actual situation at the particle interface than other contact models. Of course, not a single or typical model could be adapted to the characterization of the mechanism of the double layer of silicon–aluminum-phase materials in mixed-medium contact; some model assumptions defects could not well represent the complex realistic results [20,21]. Although the double-layer theory had seen considerable development in the field of metal–solution interfaces [22,23,24,25], no research had been performed to clarify the electrostatic layer change mechanism at the interface of the solid–liquid–solid mixed medium involved in this study.

To better clarify the direction of a breakthrough, during the advancement of recent model studies, several double-electric-layer theories have been used to help explain the water content and migration behavior in permafrost [26]. And it also has a certain development in the field of soil [27,28]. These mechanistic models have similar insights in exploring the consolidation behavior of colloidal flocculation. The charged properties of soil colloids will induce ion attraction. And the adsorption and stabilization of colloids increases when the charge increases. These findings were partly scientific, but to focus on these points only was insufficient; a deeper ion intervention situation was needed. Some studies explore the interaction between some metal ions and soil particles on particle microstructure; alkali metal ions exert their roles through different mechanisms, and these changes have significant implications on the physical and mechanical properties of the soil. Impact laws were not often present in a linear form [29]. Research in this area deserve further exploration. It is predictable that ion intervention changes the surface charge, double-layer thickness and chemical bonds between the soil particles, thus affecting their interaction. These situations can cause changes in soil particle microstructure, such as the adjustment of particle morphology, which ultimately change the mechanical behavior and stability of the soil.

In light of this, the present study characterized and elucidated the process of double-layer formation between the particle phase and liquid phase of clay and solid waste materials through the lens of the double-layer theory while proposing a relevant hypothesis. Additionally, by further analyzing ion-influenced electrostatic-layer mechanisms across various studies, this research investigated a rational approach to solid-waste base-clay stabilization. The theoretical exploration of the entire microscopic mechanism system was fundamentally significant for the reasonable adjustment of the solid–liquid–solid double layer of the silicon–aluminum phase, providing new ideas. According to numerous existing reports, the incorporation of multi-valent cation additives could be more effective in favor of the dissociation of silicon–aluminum phase molecules [30,31]. Consequently, we proposed a promising strategy that employed alkali-activated technology as a primary activation method while focusing on low-activity waste soil and other silicon–aluminum-phase materials for targeted ion selection and addition to investigate stabilization mechanisms based on ion activation in relation to double-layer dynamics.

Further, a variety of methods were employed to assess the validity of the characterization model and verification techniques, including orthogonal experimental analysis, strength test, X-ray diffraction (XRD), and microscopic analysis. This research would contribute to developing more environmentally sustainable strategies for the disposal of clay–solid waste–lithium slag and held significant guiding value for the development of soil stabilization technologies and solid waste treatment methodologies.

## 2. Establishment of Silicon–Aluminum-Phase Solid–Liquid–Solid Double-Layer Model and Solid-Waste Base-Clay Stabilization Method

### 2.1. Characterization of Solid–Liquid–Solid Non-Uniform-Diffusion Double-Layer Model Between Silicon–Aluminum Particles in Mixed Medium

A series of studies had confirmed that the double-layer mechanism had certain adaptability in characterizing the ion exchange and variation at the phase interface [32]. Similarly, as studies had shown that the compressible behavior of clay was controlled by the double layer formed around clay particles, this was one of the many expressions of using the double-layer theory to analyze and predict the interface behavior of soil particles. Taking silicon–aluminum-phase molecules as the main body of the material as an example, such as clay, lithium slag, slag, etc., the surface of the particles was negatively charged [33]. According to the principle of equilibrium attraction, cations (such as sodium ions, potassium ions, etc.) and polar water molecules would be attracted to the surface of the particles nearby [34,35]. Therefore, a “double-layer sandwich structure” could be characterized [36]. However, unlike the various theoretical models that had been described, the interface situation between silicon–aluminum-phase particles in a mixed medium was complex and involved many factors, requiring additional hypothesis, which led to the inapplicability of traditional models [37,38].

Among them, four aspects were particularly important. The first was that the mixing, diffusion, and adsorption of ions in the solution were changing and nonlinear, with a multifaceted impact [39]. The second was that the ions in the medium could not be simplified by physical means; they were three-dimensional and had a volume [40,41]. The third was that not only electrostatic forces but also chemical bonding forces and molecular thermal motion existed in the mixed medium, and the overall effect was that the attraction and repulsion phenomenon was multi-directional and non-uniform [42,43,44]. Fourthly, ions in the process of mutual attraction were not only affected by the main pairing ions but also attracted other free compensating electrons, which were not simply present and occupied volume but were affected by quantity and concentration [45,46]. Therefore, through analysis, the existing models had not been able to propose a more comprehensive adaptive model for the silicon–aluminum-phase solid–liquid–solid mixed medium. Based on this, a new conjecture model was formed by supplementing the following assumptions:

1. The outer side of the particle core (meaning the outside of silicon–aluminum-phase core interface) included both inner and outer double-sided Helmholtz planes (meaning internal adsorption surface and external non-specific adsorption surface, and inside of them can be seen as a stern layer), and at the same time, the ion movement within the layer was non-uniform (meaning an asymmetrical diffuse layer outside of the external non-specific adsorption surface).
2. Some of the anions and cations in the liquid phase would attract partially coordinated compensating ions.
3. In the solid–liquid mixed system, the double layer of the particles was influenced by ion–water complexation and hydration and ion solvation, and the attraction and repulsion between ions were non-uniform.
4. Simultaneously, the effects of adsorption force, electrostatic force, and chemical bonding force were considered.

**Figure 2:**
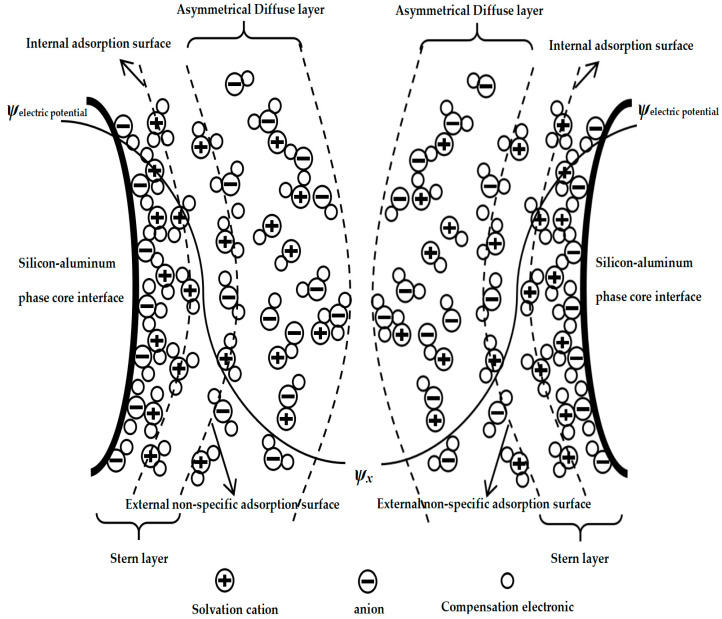
Solid–liquid–solid double-layer inhomogeneous diffusion model of silica–aluminum phase (SAP-SLSDLM).

Therefore, the non-uniform-diffusion double-layer model structure of the silicon–aluminum-phase particles in the mixed medium would be as shown in Figure 2 below.

Figure 2 shows the solid–liquid–solid double-layer inhomogeneous diffusion model of silica–aluminum phase (SAP-SLSDLM), and some special specific conditions could be further characterized. Specifically, let us assume the situation: the inner adsorption layer could allow for the adsorption of both cationic and anionic ions, while different charges or ions could be stabilized in the inner adsorption layer by multiple forces. Additionally, in the outer diffusion layer, there was attraction and repulsion between different ions, which would result in crystallization, which could wrap and attract free ligand ions from the outside in to wrap the inner particle core [47,48]. Furthermore, the variation in interlayer thickness was non-uniform due to differences in hydrogen bonding [49].

**Figure 3:**
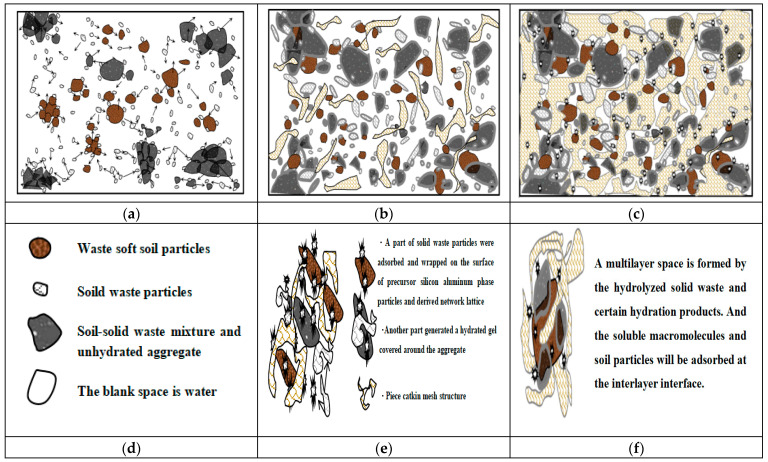
Analysis of the stabilization process based on the double-electric layer-mechanism–ion excitation modification: (**a**) the early stage of dissociation(Arrows represent the behavior of the outward-diffusion decomposition); (**b**) the continuous hydration reaction process of the mixed substances; (**c**) the stabilization state of the mixed medium; (**d**) the meaning of matter; (**e**) the state of the reactive substance; (**f**) aggregation and adsorption.

In the early stage of dissociation, the silicon–aluminum-phase particles were composed of numerous dissociable small molecular clusters, some of which could participate in hydrolysis to generate ionic clusters for decomposition while selectively adsorbing surrounding ions to maintain system equilibrium. Additionally, the cations associated with the silicon–aluminum tetrahedra or octahedra exhibited instability and might be occupied by various cations or even replaced by others, thereby providing a foundation for the utilization of certain solid wastes. Consequently, we had developed a deconstruction strategy that employed a combined excitation, that is, introducing calcium-based stabilizing agents containing a defined quantity of Ca^2+^ ions in an alkaline medium [5]. This approach promoted the continuous formation of insoluble silicate and aluminosilicate hydrolysis gels and crystallization precipitates, resulting in a compact three-dimensional porous network that filled voids and densified the soil material, and enhancing its overall stability and density. Based on this framework, a hypothetical microscopic scenario was presented; the stabilization and consolidation changes within the entire system following hydration would be illustrated through three types of the theoretical analytical diagrams, as shown in Figure 3.

Based on the analytical results, this strategic mechanism employed a series of physical adsorption and chemical interaction processes to modify the engineering properties of silicon–aluminum-phase particles within the mixed system, particularly by impelling the dissociation and polymerization reactions of precursor silicon–aluminum units. This was evidenced by the selective attraction of sodium ions in the diffusion layer, leading to compressive deformation and disassembly of the original molecular structure at the interface between silicon–aluminum-phase particles and resulting in partial release of silicon–oxygen and aluminum–oxygen structural units into the liquid phase for subsequent replacement and hydrolysis reactions [50]. In addition, calcium ions continuously generated calcium hydrates during the hydration process with soil particles and solid waste materials, wrapping and covering the surrounding unreacted aggregate particles with film-like, net-like, or clump-like calcium hydrates. This action gradually reduced interfacial double-layer distances between phases while enhancing intermolecular attractions, thereby drawing agglomerates closer together and compressing them into insoluble aggregates [51]. In this way, the thickness of the double-layer ion film was reduced through ionic interactions, intermolecular forces were strengthened, and the molecular activity within the silicon–aluminum phases was stimulated. Condensation among colloidal aggregates was promoted as they approach one another, ultimately yielding a stable and dense solidified soil material with enhanced engineering properties.

### 2.2. A Stabilization Method for Solid Waste Based on Ion Pair Interaction Mechanism Affecting the Double Layer of Clay

The establishment of the solid–liquid–solid double-layer inhomogeneous diffusion model of silica–aluminum-phase (SAP-SLSDLM) theoretical model and subsequent analysis of its mechanisms indicated that the amount of hydration cations in the modified soil itself was small. When the soil was in a high moisture state or upon contact with water, leading to hydrolysis, the hydration reaction rate of the dissociated and diffused cations was relatively high. These cations at the liquid–gas interface were readily hydrated, resulting in an increased thickness of the diffusion layer between particles [52]. This thickening led to a higher electrical potential difference among particles, which enhanced repulsive forces between them. Consequently, this enhanced blocking effect promoted dispersion during molecular motion and manifests as a degree of water solubility—aligning closely with observed behaviors when soils dissolved in water.

To improve the engineering properties of low-activity silicon–aluminum-phase materials, it was essential to first clear that their surface charge was constant. The basic parameters could be confirmed through physicochemical analysis, including X-ray diffraction (XRD) detection and basic physical property measurement. Furthermore, in order to promote the consolidation stability of the mixed system, it was necessary to reduce the thickness of the double layer of the entire system, facilitating particle aggregation. Quantitative addition of ions should be based on specific ion species and concentrations for optimal economic compatibility. Specifically, when high-valence ions were introduced into a negatively charged silicon–aluminum-phase system, it was crucial to consider both hydrolysis degree and hydration capacity as key indicators for regulating the dispersion or aggregation of silicon–aluminum-phase particles within the mixed system. Additionally, for the characteristics of insufficient hydration products of low-activity silicon–aluminum phase, beneficial transformations were required by adding strong skeleton components, such as calcium-containing compounds that were easily hydrolyzed [53]. The results from model mechanisms indicated that the concentration of ions was nonlinear to the regulation of the thickness of the diffusion double layer. Thus, appropriate matching and experimental validation were required to determine optimal addition amounts, facilitating soil particle aggregation and achieving effective consolidation and stability.

**Figure 4:**
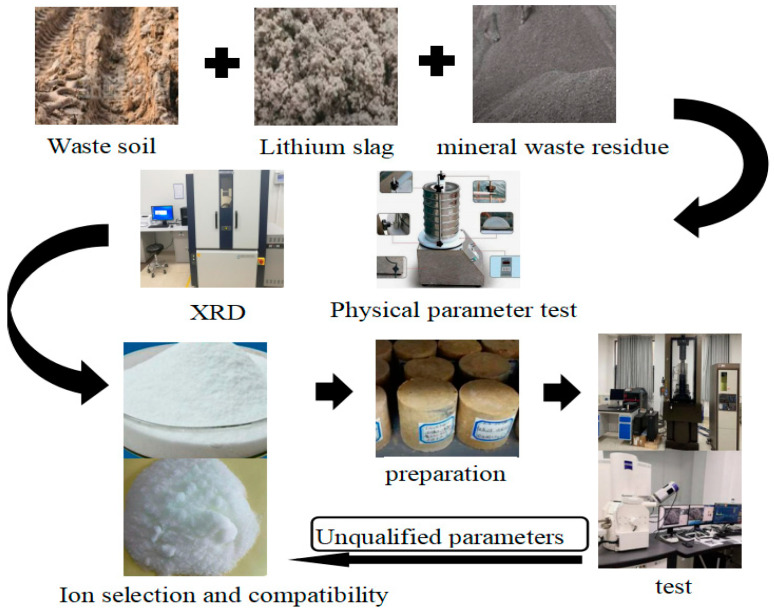
Solid waste foundation-clay stabilization method based on the influence mechanism of ions on double electrolayer.

In conclusion, the comprehensive modification process and regulatory mechanisms would be further elucidated, with the solid-waste base-clay stabilization method informed by the ion-pair double-layer influence mechanism presented as shown in Figure 4.

## 3. Synergistic Treatment Stabilization Experiment and Analysis

### 3.1. Experiment Materials and Equipment

The experiment-undisturbed clay was taken from a place in Changsha, Hunan, China (28°13′52.4″ N, 112°56′0.4″ E), and the soil was relatively soft and loose, with a yellow–brown appearance. The experimental solid waste lithium slag was the waste material after secondary lithium extraction, which came from Yichun, Jiangxi, China (27°49′1.1″ N, 114°24′40.5″ E) and was moist and loose; its appearance was grayish–white powder after drying. Materials came from multiple batches of samples, and the average of the three groups was selected as the final attribute data. The basic physical properties and chemical composition were determined through testing and X-ray diffraction (XRD) analysis, with the results shown in Table 1 and Table 2, respectively. And the test method of this experiment involves an electronic universal testing machine (WDW-100C) from China Wuhan-Glamo (Wuhan, China), an X-ray diffractometer (D8 Advance) from Germany-Bruker (Ettlingen, Germany), and an emission-scanning electron microscope (S-4800) from Japan-Hitachi (Hitachi, Japan).

Among them, calcium oxide (the purity degree was 99%, Guangzhou, China), NaOH (the purity degree was 96%, Tianjin, China), and CaCO_3_ (the purity degree was 99%, Shandong, China) were all AR-analysis-pure powder, and the test water was taken from the laboratory.

Furthermore, the unconfined compressive strength test was conducted using the WDW-100C electronic universal testing machine, and the X-ray diffraction (XRD) analysis was performed using a Bruker D8 Advance X-ray diffractometer (from Germany-Bruker (Ettlingen, Germany)), with a 2θ measurement range of 10° to 80°, a scanning rate of 2 (°)/min, and a voltage of 40 kV. Microscopic analysis was carried out using a Hitachi S-4800 scanning electron microscope (from Japan-Hitachi (Hitachi, Japan)). At the same time, a drying oven, electronic scale, 80 mm high and 39.1 mm diameter test mold, plastic fresh-keeping bag, measuring cylinder, and mixing rod were used.

### 3.2. Test Scheme

In order to explore the validity of the theoretical mechanism, a variety of experiments were designed and carried out, including orthogonal experiment, unconfined compressive strength test, and X-ray diffraction (XRD) and scanning electron microscope (SEM) analysis. The number of experimental trials was sufficient to ensure reproducibility, because the average of the results from multiple replicate experiments was chosen as the final data. Meanwhile, the error of the experimental results was controlled, with a 5% margin of error.

#### 3.2.1. Orthogonal Experiment and Verification Analysis

Four factors were designed: lithium-slag-mixing ratio (the percentage of the mass of the added material to the mass of the test dry soil, the same below), calcium-oxide-mixing ratio, composite-activator (CaCO_3_ and NaOH)-mixing ratio, and their mixing ratio (CaCO_3_ and NaOH mass ratio), with each factor setting at four levels. Using L16(44) orthogonal table, the orthogonal experiment was conducted with unconfined compressive strength at 7 days and 28 days as the test index to analyze the effect of each factor, as shown in Table 3.

The results of the orthogonal experiment were shown in Table 4, where *K*_1_, *K*_2_, *K*_3_, *K*_4_ and *R*_1_, *R*_2_, *R*_3_, *R*_4_ represented the sum of unconfined compressive strength under different factor levels at 7 days and 28 days ages, respectively. The orthogonal tests using the L16 (44) orthogonal table under 4 factors. At the same time, *K*_1_, *K*_2_, *K*_3_, and *K*_4_ represent the sum of the four groups at the level of four changing factors at 7 days of age. And *R*_1_, *R*_2_, *R*_3_, and *R*_4_ represent the sum of the four groups at the level of four variable factors at 28 days of age. Among them, the subscript number represents the number sequence corresponding to the four influencing factors in the definition, and the number determines that the corresponding factors belong to the data within the corresponding rows.

**Figure 5:**
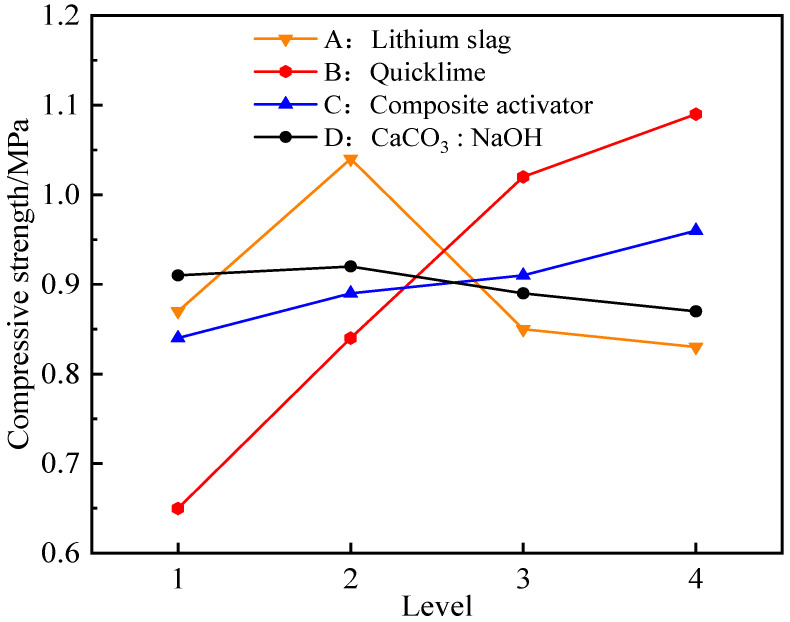
Compressive strength changes in the level of various factors at 7 days.

Through the range analysis of Table 3, it could be seen that the influence of various factors on the 7 days and 28 days compressive strength of the composite activated lithium slag-based solidified soil was in the following order: quicklime incorporation ratio, lithium slag incorporation ratio, composite activator incorporation ratio, calcium carbonate to sodium hydroxide content ratio. For a more intuitive analysis, the trend of average compressive strength changes in the level of various factors at 7 days of age was drawn, as shown in Figure 5.

According to Figure 5, when the lithium slag incorporation was relatively small, the compressive strength increased with the increase in the incorporation ratio. For example, when the incorporation ratio increased from 14% to 16%, the strength increased by 19.54%. But, when the incorporation ratio was 18% and 20%, the compressive strength decreased, so the ideal addition ratio of lithium slag in this experiment was 16%. In addition, as the quicklime incorporation ratio increased from 1% to 2% and to 3%, the compressive strength of the solidified soil was significantly enhanced, with growth rates of 29.34% and 22.09%, respectively. However, from 3% to 4%, the compressive strength increased, but the growth rate decreased only 6.11%. Compared with some studies, the addition of calcium promotes gelation and formation, which is almost linear, and the amount of addition affects the size of the product [54]. It was believed that when the CaO incorporation ratio was relatively small, the potential activity of the silicon–aluminum-phase material could not be effectively stimulated. Conversely, as the CaO incorporation ratio was further increased, the initial hydration reaction of the silicon–aluminum-phase material would be inhibited, and a large amount of unreacted free CaO would form weak structures in the mixture, thereby affecting the compressive strength characteristics of the solidified soil. Meanwhile, the influence of the mixing ratio of CaCO_3_ to NaOH on the compressive strength could also demonstrate the accuracy of the theoretical model mechanism. For example, when the ratio increased from 0.5 to 1.0, the compressive strength increased, but as the ratio continued to increase, the strength gradually decreased. The analysis indicated that when the calcium carbonate-to-sodium hydroxide ratio was too large, excessive ion injection was not conducive to stimulating the activity of silicon–aluminum-phase materials. At the same time, the surplus ions hindered the hydration contact area of silicon–aluminum-phase molecules, thereby inhibiting the formation of strong hydration products. This result was of great practical significance for the regulation of ion-controlled dosage.

#### 3.2.2. Unconfined Compressive Strength Test

Based on this, the undisturbed clay was further designed as the experimental control group, and three groups of comparative tests were made in parallel. The experimental design was shown in Table 5.

**Figure 6:**
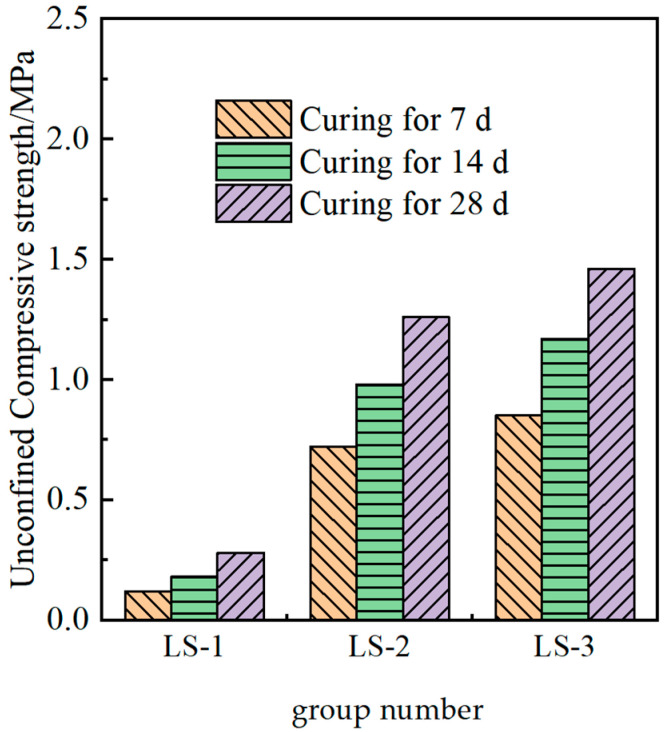
Strength of cured soils with different functions of admixtures.

The measured strength of the solidified soil under different ion additions is shown in Figure 6.

It could be seen from Figure 6 that the effect of adding lithium slag alone in the test soil was not obvious, and its activity was poor, making it impossible to form a skeleton structure with strength. Compared with the findings in some studies, lithium slag is suitable for auxiliary addition, but excessive lithium slag would adversely affect the mechanical properties of the material. Lithium slag performs well after mixing with some excitation materials [13]. Compared with the LS-1 group, the strength of the LS-2 and LS-3 groups was significantly improved, demonstrating that the potential cementitious activity of lithium slag could be stimulated under the action of composite ions, and the strength of the modified material could be enhanced. In addition, the increase in calcium ions from LS-2 to LS-3 led to a lower strength, which was consistent with the theoretical mechanism described.

#### 3.2.3. X-Ray Diffraction (XRD) Phase Analysis

**Figure 7:**
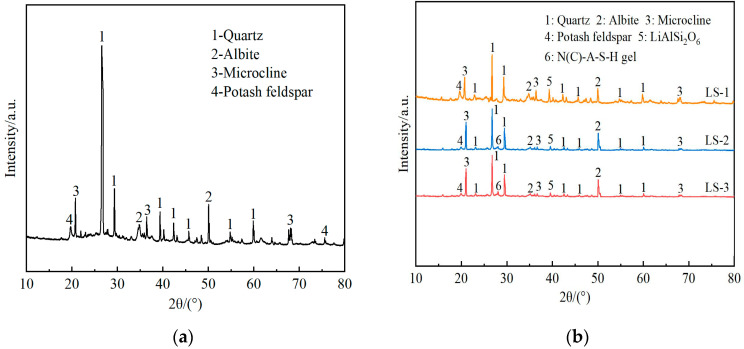
The X-ray diffraction (XRD) patterns of undisturbed clay and solidified soil with different functions of admixtures: (**a**) undisturbed clay; (**b**) solidified soil samples with different functions of admixtures.

The X-ray diffraction (XRD) patterns of undisturbed clay and solidified soil samples with different functions of admixtures after curing for 28 days were shown in Figure 7.

From the analysis of Figure 7a, the main crystalline phases of undisturbed clay were quartz, albite, and potassium feldspar, with quartz being the most abundant. According to LS-1 in Figure 7b, when only lithium slag was added, except for the appearance of a small amount of lithium pyroxene, the rest of the crystal phases were basically the same as the undisturbed clay. That was to say, there was no new material production in the process of only adding lithium slag, and the soil performance was not significantly improved.

By observing Figure 7b LS-1 and LS-2 in the 2θ area of 25~30°, it can be seen that with the incorporation of complex ions, the product represented by the number 6 and the diffuse “steamed bread” like broad peak appeared in the Figure 7b LS-2 at the 2θ area of 25~30°. This study showed that the “steamed bread” peak was related to the newly generated N-A-S-H gel and C-A-S-H gel [55], which was because the Si-O bond and Al-O bond in the silicon–aluminum-phase oxide were more prone to break in an alkaline environment, leading to an increase in the hydration reaction rate. As a result, the network structure was disrupted, and calcium aluminosilicate hydrate (C-A-S-H) gels were regenerated through repolymerization. Moreover, the cations in the system and Na^+^ in the alkali solution were ion-exchanged through charge balance, generating sodium aluminosilicate hydrate (N-A-S-H) gels [56]. Among them, C-A-S-H gel had a dense structure similar to hydrated calcium silicate gel (C-S-H), and N-A-S-H gel had a zeolite phase structure [36], which was beneficial for the development of the strength of solidified soil. At the same time, compared with the peaks of undisturbed clay and LS-1, the peaks associated with quartz, sodium feldspar, and spodumene were reduced. The main reason for this was that some substances in the silica–alumina phase were hydrolyzed, which increased the cohesion of solidified soil particles. The flocculated colloidal structure formed thereby reduced the reflection of incident rays.

In addition, comparing the peak curves of LS-2 and LS-3 revealed that there was a difference in the “steamed bread” shape’s continuous diffuse peak package in the 2θ area of 25~30°. The peak heights in LS-2 were lower than those in LS-3 to a certain extent, indicating that more gel-phase products were generated in LS-3 solidified soil, presumably due to the better synergistic effect of the two. In contrast, the concentration of Ca^2+^ ions in the slurry of LS-2 increased, but the (N) C-A-S-H gel product was reduced. Compared with similar results, excessive calcium ion content will also affect the entire hydration process, which will lead to the dissociation of more acid ions in solution, which in turn will worsen the structural damage [57]. It was analyzed that the polymer formed by the direct condensation of some of the reaction ions encapsulated the silicon–aluminum-phase particles that had not yet participated in the reaction, hindering further hydrolysis of the silicon–aluminum materials. As a result, the reflected (N)C-A-S-H peak was decreased, and its structural strength was lower than that of LS-3. In addition, the results of the unconfined compressive strength test verified this point.

#### 3.2.4. Microstructure Analysis

**Figure 8:**
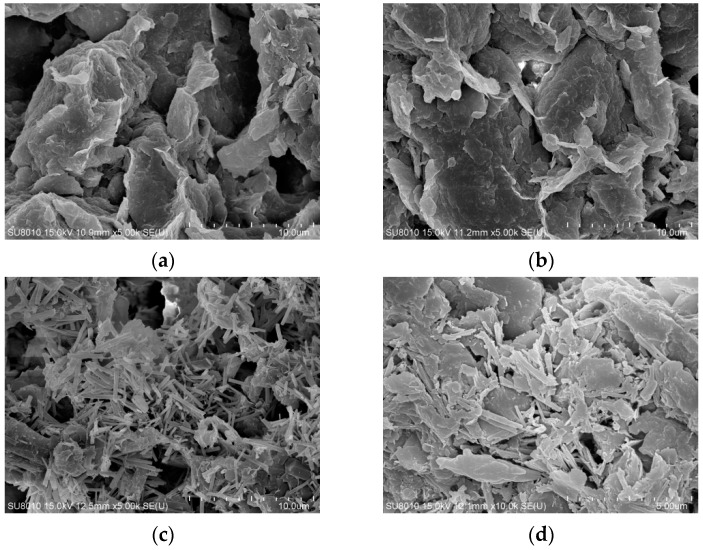
The scanning electron microscope (SEM) images of undisturbed clay and solidified soil samples with different functions of admixtures: (**a**) undisturbed clay, ×5000; (**b**) LS-1, ×5000; (**c**) LS-2, ×5000; (**d**) LS-3, ×10,000.

Scanning electron microscope (SEM) images of undisturbed clay and solidified soil samples cured for 28 days with different functions of admixtures under scanning electron microscopy test were shown in Figure 8.

In Figure 8a, the undisturbed clay was characterized by a flaky structure and irregular blocky and layered structure, which were superimposed into uneven clumps with some spatial faults. There were many holes and pores between the flocs, and small flocs were connected between particles, resulting in larger pores within the structure, so the strength is low. The microstructure of the LS-1 sample in Figure 8b was similar to that of the undisturbed clay. Some short needle-column lithium slag particles were relatively dispersed and wrapped in soil particle flocs, with large structural pores, and there were still holes and interlayer voids in the mixed material. It can be seen that a small number of lithium slag particles were adsorbed by some clay particles, and filamentous colloids were generated, showing that a small amount of water molecules in the clay particles promoted the formation of a “diffusion double layer structure” between the particles, resulting in a slight hydrolysis reaction and slight agglomeration phenomena.

As illustrated in Figure 8c, some short needle-column gel products were relatively dispersed and covered the surface of the soil particles, and there were still unreacted white flocs and similar hydrated calcium gel products appearing between flocs. The pores between the soil particles could not be uniformly filled by the gel phase, but there were holes and interlayer voids, and some similar filaments and colloids were attached to the surface of the rod-shaped lithium slag, obviously revealing the presence of insufficiently reacted ions. The phenomenon was basically consistent with the theoretical mechanism characterization. A similar microscopic study pointed out that the lithium slag would reduce the structural form in the early hydration or insufficient reaction, and the use of lithium slag with an appropriate content of addition and the appropriate mixing improves microstructure at later ages [11]. Compared with the LS-3 group, the ion content had a certain increase, which was found to be due to the excessive addition of calcium ions, the unhydrated ions attached to the surface of the particles, conversely hindering further hydrolysis of the silicon–aluminum phase. This also explained the microscopic manifestation of reduced flocculation caused by the increase in the thickness of the electric layer, resulting in the structure of the LS-2 group being less compact than the LS-3 group.

For LS-3 (Figure 8d), under the observation of higher magnification, the microstructure was denser than other groups, and the number of hydration products increased. Meanwhile, a large number of white flocculent cementation products were adhered to the particles, effectively cementing the various products together and wrapping around the soil particles. The cementing materials overlapped with each other, encapsulated the soil particles, and fully filled the voids in the sample, thus forming a network-like mixed framework structure and presenting a large flocculent shape of the wave layer. The microscopic appearance confirmed the validity of the theoretical mechanism.

In summary, the SEM images of Figure 8c LS-2 and Figure 8d LS-3 show and illustrate robust, network-like structures compared to the undisturbed clay in Figure 8a, of the flocculation-group-like needle bar overlapping with each other at some external surface. Moreover, when comparing the SEM images of Figure 8c LS-2 and Figure 8d LS-3, it can be seen that Figure 8c LS-2 increases the calcium ion incorporation parameters but shows an image of an incomplete compact structure; some hydrated calcium catkins attach to the particle surface, and some obvious rod-shaped particles were interlaced in the flocs. Differently, the calcium ions in Figure 8d LS-3 were less than in Figure 8c LS-2. In Figure 8d, the rod particles in LS-3 change to flat catkins, with a large and tight distributed area, which indicates that LS-3 has more adequate decomposition and reaction. Meanwhile, the tighter SEM structure also showed a significant increase in the compressive strength of LS-2 and LS-3, which signifies the success of the stabilization method.

## 4. Discussion and Verification

The model mechanism demonstrated good applicability in characterizing the interfacial mechanism of silica–aluminum-phase mixing. The results of the strength test and microscopic analysis showed that sodium ions had a significant effect on the non-uniform diffusion double layer between mineral particles in the liquid–solid mixed system. Under the premise of water involvement, the addition of sodium ions as high-valence ions to the system could be beneficial to reduce the thickness of the diffusion double layer between solid waste particles and soil particles. Furthermore, the attraction between particles was enhanced, which was conducive to the agglomeration of various molecules. At the same time, the addition of calcium ions had two improvement effects on soil particles with poor engineering performance. On the one hand, the cation exchange occurred as calcium ions replaced lower-valence ions in the soil, while the calcium ions that did not exchange participated in hydration to form a gel, making the particle space trap close. On the other hand, the absorption of calcium ions by particles in the mixed medium squeezed the diffuse double layer of soil particle colloids, resulting in a decrease in the thickness of the double layer between particles, and the granules gradually agglomerated and shrunk.

Moreover, the experimental results indicated that the lithium slag itself did not have cementitious activity and had poor hydraulicity. When the solid waste was compounded with the low-strength cohesive soil to be modified, a certain synergistic effect was observed, and the compressive strength of the samples was significantly enhanced after synergistic modification treatment. Comparing the results of the LS-2 and LS-3 groups, with the amount of added calcium ions as a variable, it was observed that as the addition amount was increased, the strength-forming process of the solid waste lithium slag group was inhibited, leading to a decrease in the final sample strength. This phenomenon was more in line with the theoretical model mechanism analysis results. It was analyzed that there might be a large number of unreacted calcium-ion-containing components in the particles, which would form weak structures in the mixture, causing the enrichment and extrusion of ions on the surface of the silicon–aluminum-phase particles, and losing the space to react with other substances. Consequently, the original part of the cations that could participate in the hydration reaction were unable to react with the coordination ions, and the thickness of the diffusion layer increased, adversely affecting the strength of the samples. The experimental results defined the correctness of the theoretical model excitation idea, and the enhancement effect of strength demonstrated the effectiveness of the theoretical method.

Based on the results of the research, a practical approach would be elaborated. Compared with the traditional way, the proposed model mechanism was more conducive to the demand of material modification, and it was a collaborative treatment method combined with waste resources. These findings will be able to be used in some construction projects, and there was a huge amount of excavation in these projects. Unlike some traditional resource treatment methods in construction projects, this method avoids the excavation and transportation methods of traditional projects and saves money on extra external transport and processing costs. For some excavated clay with poor engineering properties, some waste resources can be used in clay to change their performance, and the new materials can be used for some pit back filling and laying. Moreover, based on the results of theoretical mechanism, we consider the needs of market and application. Compared with some studies choosing expensive materials [58], more appropriate sodium ion sources and calcium ion sources were used in ion selection. These ways were more novel than traditional methods and more practical and economical than results from existing studies.

## 5. Conclusions

In light of the engineering challenges associated with soil excavation and utilization, as well as the extensive disposal and treatment of solid waste, this study adopted an integrated theoretical and practical approach to develop theoretical models and implement experimental applications. Additionally, the validity of the proposed theoretical model and the micro-stabilization mechanisms for solid waste were assessed through various methodologies, including orthogonal experiment analysis, unconfined compressive strength test and comparison test, X-ray diffraction (XRD) phase analysis, and microstructure analysis. The following conclusions could be drawn:

1. A dual-layer model of solid–liquid–solid for the silicon–aluminum phase in the mixed medium was proposed to characterize the influence of ions on the interfacial dual-layer of particles. Substances based on sodium and calcium ions could reduce the thickness of the diffusion double layer between silicon–aluminum phases in mixed soil media; however, excessive ion concentrations might inhibit particle agglomeration. The relationship between ion selectivity and changes in double-layer thickness was nonlinear, indicating that their effects were not governed by a constant law.
2. The results of the orthogonal experiment and unconfined compressive strength test elucidated the stabilization mechanism and validated the effectiveness of the solid-waste-co-processing method. Comparative test findings indicated that while lithium slag did not exhibit potential for strength enhancement when mixed with clay, it demonstrated significant synergistic effects upon ion stimulation, thereby achieving effective stabilization of soft soil.
3. The incorporation of composite ions significantly influenced the strength of the mixed material. An increase in calcium ion concentration correlated with a reduction in strength. The experimental data aligned closely with the predictions made by the model mechanism.
4. Microscopic analysis using the X-ray diffraction (XRD) and the scanning electron microscope (SEM) revealed that the stabilized soil resulting from synergistic treatment exhibited a compact and dense structure. A substantial amount of C(N)-A-S-H gel was generated within the hardened soil, with some directly filling excess voids between soil particles to form aggregates. The remaining gel contributed to the spatial structural system of the hardened soil through interlocking and bonding mechanisms. In contrast, the microscopic structure of the control group indicated that unreacted white floc products were still present around particle layers, leading to compression voids in the framework structure—an important factor contributing to strength reduction. The observed microstructural characteristics aligned well with predictions from theoretical model analysis.

Building on the aforementioned conclusions, a comprehensive resource utilization approach could be implemented for the substantial quantities of existing solid waste by employing the theoretical model and mechanisms to guide the addition and modification strategies for various treated soil resources.

Based on the results obtained in the research, we found that the factors considered in the research had some limitations, and the tests more in line with the practical application environment were limited by the test conditions and site funding. Therefore, this research deserves to be considered and advanced by more factors, such as considering different temperatures, different humidity changes, and different application scenarios. We believe that the consideration of these factors will lead to more results, which will promote the more accurate prediction of some waste additions in the direction of material improvement.

## Figures and Tables

**Table 1:** Basic physical properties of soft soil.

Maximum Dry Density/(g·cm^−3^)	Optimal Moisture Content/%	Liquid Limit/%	Plastic Limit/%	Liquidity Index	Plasticity Index
1.78	22.5	34.2	21.6	0.56	12.6

Note: % is mass fraction; these data were obtained from the average of results from multiple replicate experiments, with a 3% margin of error.

**Table 2:** Main chemical components of soft soil and lithium slag.

Material	Mass Fraction/%							
	SiO_2_	Al_2_O_3_	CaO	Fe_2_O_3_	MgO	SO_3_	Alkali Content	Loss on Burning
Soft soil	63.24	20.24	2.49	4.51	1.39	-	-	-
Lithium slag	42.50	22.40	8.40	2.60	1.00	9.20	8.60	3.00

Note: % is mass fraction; these data were obtained from the average of results from multiple replicate experiments, with a 3% margin of error.

**Table 3:** Table of factor and level for orthogonal experiment.

Factor	Level			
	Lithium Slag/% (A)	Quicklime/% (B)	Composite Activator/% (C)	CaCO_3_:NaOH (D)
1	14	1	0.8	0.5
2	16	2	1.2	1.0
3	18	3	1.6	1.5
4	20	4	2.0	2.0

Note: % is mass fraction.

**Table 4:** Results of orthogonal experiment.

Number	Factor				Compressive Strength/MPa	
	A	B	C	D	7 Days	28 Days
A_1_	1 (14)	1 (1)	1 (0.8)	1 (0.5)	0.59	0.73
A_2_	1 (14)	2 (2)	2 (1.2)	2 (1.0)	0.89	1.02
A_3_	1 (14)	3 (3)	3 (1.6)	3 (1.5)	0.93	1.25
A_4_	1 (14)	4 (4)	4 (2.0)	4 (2.0)	1.08	1.48
A_5_	2 (16)	1 (1)	2 (1.2)	3 (1.5)	0.76	1.18
A_6_	2 (16)	2 (2)	1 (0.8)	4 (2.0)	0.82	1.25
A_7_	2 (16)	3 (3)	4 (2.0)	1 (0.5)	1.30	1.84
A_8_	2 (16)	4 (4)	3 (1.6)	2 (1.0)	1.27	2.12
A_9_	3 (18)	1 (1)	3 (1.6)	4 (2.0)	0.65	1.12
A_10_	3 (18)	2 (2)	4 (2.0)	3 (1.5)	0.86	1.37
A_11_	3 (18)	3 (3)	1 (0.8)	2 (1.0)	0.92	1.52
A_12_	3 (18)	4 (4)	2 (1.2)	1 (0.5)	0.97	1.61
A_13_	4 (20)	1 (1)	4 (2.0)	2 (1.0)	0.59	1.12
A_14_	4 (20)	2 (2)	3 (1.6)	1 (0.5)	0.78	1.24
A_15_	4 (20)	3 (3)	2 (1.2)	4 (2.0)	0.94	1.59
A_16_	4 (20)	4 (4)	1 (0.8)	3 (1.5)	1.02	1.68
*K*_1_	3.49	2.59	3.35	3.64		
*K*_2_	4.15	3.35	3.56	3.67		
*K*_3_	3.40	4.09	3.63	3.57		
*K*_4_	3.33	4.34	3.83	3.49		
*R*_1_	4.48	4.15	5.18	5.42		
*R*_2_	6.39	4.88	5.40	5.78		
*R*_3_	5.62	6.20	5.73	5.48		
*R*_4_	5.63	6.89	5.81	5.44		

Note: These data were obtained from the average of multiple replicate experiments, with a 5% margin of error.

**Table 5:** Comparative experimental design.

Number	Material Type and Mixing Ratio			
	Lithium Slag/%	CaO/%	CaCO_3_/%	NaOH/%
LS-1	16			
LS-2	16	6	1	1
LS-3	16	4	1	1

Note: % is mass fraction; LS-X means the group of the lithium slag with different additions, and X is group number, including 1, 2, and 3.

## Data Availability

The data presented in this study are available on request from the corresponding author.

## Abbreviations

The following abbreviations are used in this manuscript:

SEM	Scanning electron microscope
XRD	X-ray diffraction
SAP-SLSDLM	Solid–liquid–solid double-layer inhomogeneous diffusion model of silica- aluminum phase
